# Patients’ Experiences of Diagnostic and Therapeutic High-Resolution Endoscopy in Treating Anal Squamous Intraepithelial Lesions: A Qualitative Study

**DOI:** 10.3390/diagnostics15172205

**Published:** 2025-08-30

**Authors:** Peter Borch-Johnsen, Hanna Dubois, Peter T. Schmidt, Jonas Nygren, Gail Dunberger

**Affiliations:** 1 Department of Medicine, Ersta Hospital, 116 30 Stockholm, Sweden; 2 Department of Medical Sciences, Uppsala University, 751 05 Uppsala, Sweden; 3 Department of Clinical Science and Education, Södersjukhuset, Karolinska Institutet, 118 83 Stockholm, Sweden; hanna.dubois@ki.se; 4 Theme Cancer, Karolinska University Hospital, 171 76 Stockholm, Sweden; 5 Department of Surgery, Ersta Hospital, 116 28 Stockholm, Sweden; jonas.nygren@erstadiakoni.se; 6 Department of Clinical Sciences, Danderyd Hospital, Karolinska Institutet, 171 77 Stockholm, Sweden; 7 Department of Health Care Sciences, Marie Cederschiöld University, 116 28 Stockholm, Sweden

**Keywords:** anal cancer, anal squamous intraepithelial lesions, early cancer detection, diagnostic acceptability, anxiety, cancer fear, high-resolution endoscopy, endoscopic treatment, human papillomavirus, patient experience, qualitative research

## Abstract

**Background:** Anal squamous cell carcinoma is a rare disease strongly associated with the human papillomavirus (HPV) and preceded by the premalignant anal squamous intraepithelial lesion (ASIL). High-resolution anoscopy (HRA) using a colposcope is considered the gold standard for detecting and managing ASIL. Despite being recommended in current guidelines for anal cancer screening, HRA availability remains limited. Although generally well tolerated, concerns about follow-up adherence persist. We have developed an endoscopic technique using high-resolution flexible endoscopes for detection, resection, and screening of ASIL. Our previous research suggests that this method is effective and gentle, but patients’ experiences of this approach remain underexplored. The aim of this study was to explore patients’ experiences of endoscopic detection, treatment, and screening of anal squamous intraepithelial lesions. **Method:** A qualitative approach was used involving semi-structured interviews and abductive qualitative content analysis. The 32-item COREQ checklist guided the reporting of the study. All participants followed a standardized protocol for treatment and follow-up. **Results:** Analysis of 16 interviews (female *n* = 7, male *n* = 9, age 19–72 years) yielded four categories: a comforting encounter in an exposed situation (with four subcategories); impact on intimate relationships (with one subcategory); living with uncertainty (with four subcategories); and physical discomfort (with two subcategories). **Conclusions:** High-resolution endoscopy is a well-tolerated and effective diagnostic and therapeutic modality for ASIL. However, the psychological impact of HPV-related conditions highlights the need for appropriate psychosocial support. These findings underscore the importance of integrating patient-centered care principles into the implementation of novel diagnostic and therapeutic technologies.

## 1. Introduction

The incidence of anal squamous cell carcinoma (ASCC) is currently low in the general population, affecting around 200 individuals per year in Sweden, but is rising [1]. The risk of developing ASCC is increased significantly in well-defined risk groups; however, despite this increased risk, these groups are not regularly screened. ASCC develops slowly from an anal squamous intraepithelial lesion (ASIL) in the pre-stage [2]. ASIL is associated with human papillomavirus (HPV) infection and the presence of condyloma. Prospective studies suggest that 9–13% of patients with high-grade squamous cell lesions (HSIL) progress to cancer within 5 years, with the highest risk of progression being among HIV-positive men who have sex with men (MSM); HIV-positive individuals; women with a history of vulvar, cervical, or vaginal HSIL or cancer; solid organ transplant recipients; and immunocompromised persons [3,4,5].

High-resolution anoscopy (HRA) is widely regarded as the gold standard for the detection, treatment, and screening of ASIL [5]. However, the procedure is performed by only a relatively small number of qualified practitioners [6]. Although HRA is generally well tolerated by patients, adherence to follow-up protocols remains inconsistent, with up to 50% of treated individuals failing to attend their recommended follow-up appointments [7]. A new method has been developed at our hospital where high-resolution (HR) flexible endoscopy is used for the detection, resection, and screening of ASIL [8].

Similarities with other endoscopic procedures exist, such as endoscopic treatments in the colon and rectum. Patients’ experiences of endoscopy in the gastrointestinal tract are often reported as being negative, and described in terms of being invasive, vexatious, and painful [9,10,11]. 

Since endoscopic treatment for ASIL is a relatively recent innovation, there is a clear need to gain deeper insight into how patients experience and perceive this therapeutic approach. ASIL is frequently associated with a high rate of recurrence, necessitating sustained surveillance and repeated treatment interventions. Understanding the patient perspective is, therefore, essential to improving clinical outcomes, enhancing adherence, and guiding the development of patient-centered care strategies.

### AIM

The aim of the study was to explore patients’ experiences of endoscopic detection, treatment, and screening of anal squamous intraepithelial lesions.

## 2. Method

**Figure 1:**
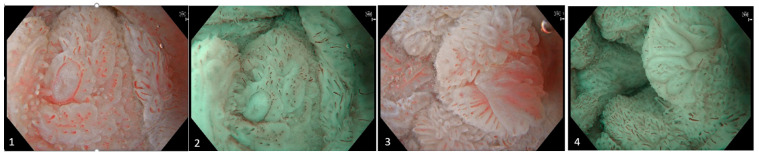
Images (**1**)–(**4**). The lesion appears nearly circumferential and is classified as ASIL (anal squamous intraepithelial lesion), with focal areas of suspected HSIL (high-grade squamous intraepithelial lesion) extending from the anorectal junction down to the anal verge. The examination was performed using an EVIS EXERA GIF-EZ1500 endoscope (Olympus Medical Systems, Shinjuku, Japan). Mucosal assessment was conducted utilizing both texture and color enhancement imaging (TXI) and narrow band imaging (NBI). Endoscopic evaluation reveals irregular and dilated intrapapillary capillary loops (IPCLs), along with areas exhibiting circular capillary patterns. Morphologically, the lesion demonstrates a combination of flat to slightly elevated villous, exophytic, and nodular features.

**Figure 2:**
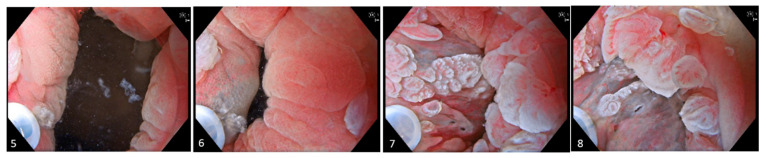
Images (**5**)–(**8**). Prior to resection, lidocaine 10 mg/mL is administered into the submucosa just above the anorectal junction on the cylinder epithelium side to create a submucosal cushion. Subsequent intermittent injections are delivered into the distal portion of this cushion to ensure comprehensive anesthetization of the targeted resection area.

**Figure 3:**
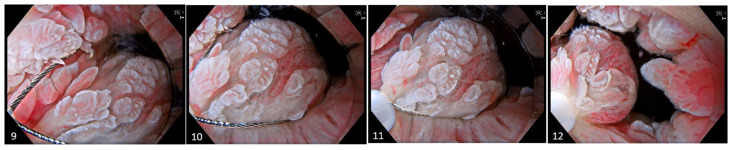
Images (**9**)–(**12**). The snare is meticulously deployed circumferentially around the target with deliberate mucosal apposition to ensure adequate tissue capture. Gradual closure of the snare is performed until tactile resistance indicates secure entrapment. Upon confirmation, the snare is slightly elevated from the mucosal surface and resection is executed under water immersion, optimizing visualization and minimizing thermal injury to surrounding tissues. For larger lesions, piecemeal resection is performed.

**Figure 4:**
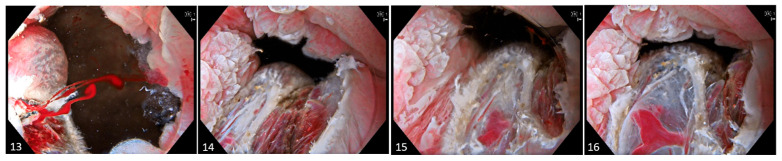
Images (**13**)–(**16**). Post-resection, the wound is carefully inspected. Minor bleeding is managed by applying targeted coagulation using the tip of the snare. Margins are assessed for residual dysplastic tissue, and if suspected, thermal ablation is performed to mitigate potential recurrence. Superficial non-bleeding vessels are left untreated as intervention is deemed unnecessary. The procedure is repeated until macroscopic radicality is achieved. In cases involving extensive lesions encompassing a substantial portion of the circumferential mucosa, segmental resection of approximately 30–40% is performed to ensure safety and preserve anatomical integrity.

Prior to therapeutic intervention, patients undergo a comprehensive diagnostic evaluation to determine the extent of suspected epithelial dysplasia. This includes a macroscopic assessment of dysplasia grade based on morphological features and vascular architecture, with particular emphasis on the classification of intraepithelial papillary capillary loop (IPCL) patterns. Bowel preparation is not required for the diagnostic examination. Previously, preoperative bowel preparation for major colorectal resections involved a liquid diet combined with 1 L of polyethylene glycol (PEG) solution, while minor resections were preceded by a 240 mL Klyx^®^ (docusate sodium/sorbito; Ferring Läkemedel AB, Malmö, Sweden) enema. However, many patients reported significant discomfort with the ingestion of the PEG solution [8]. Subsequent clinical observations have demonstrated that Klyx^®^ enemas provide adequate bowel cleansing, even for more extensive resections. As a result, the use of Klyx^®^ was standardized across all types of procedures. The procedure is described and illustrated in Figure 1, Figure 2, Figure 3 and Figure 4.

A standardized protocol for treatment and follow-up assessments was implemented and maintained throughout the duration of the study (Table 1).

A qualitative design was employed to explore patients’ experiences of endoscopic treatment for ASIL. Data were collected through semi-structured interviews and analyzed using abductive qualitative content analysis in accordance with Graneheim and Lundman’s framework [12,13]. This method enabled interpretation of both manifest and latent content, allowing for a nuanced understanding through iterative interaction between empirical data and theoretical perspectives.

### 2.1. Sampling Strategy and Settings

Men and women, aged 18 years or older, who had undergone at least one endoscopic treatment of ASIL were contacted. All participants had to understand written or spoken Swedish. In order to obtain an unbiased sample, all patients who had been treated from 1 January 2022 and onwards were invited to participate in the study. Letters of invitation were sent out between September 2022 and February 2023. Patients interested in participating were contacted by a research nurse who provided additional information about the study and gave patients the possibility to ask questions regarding any ambiguities. An appointment was then booked for an interview. The study participants could choose between a physical interview at the hospital or a digital video meeting. The interviews were conducted by two research nurses who were familiar with semi-structured interview techniques. Before the onset of the interviews, participants were once again informed about the purpose of the study, about confidentiality, and that they could discontinue their participation at any time without affecting their future care. Informed consent was obtained in writing before starting the interviews. If interviewed digitally, a recording was made after participants gave their approval and informed consent.

At the time of the study, two endoscopists at the endoscopy unit, including the first author, had been trained to perform the examination and treatment of ASIL. The endoscopic team performing the treatment included one endoscopist (nurse endoscopist or gastroenterologist) and one or two assistants (nurse or assistant nurse). A contact nurse was employed within the ASIL team to book patient appointments, answer general questions about the treatment or possible complications, and deliver certain test results. This nurse also managed logistics and participated in planning ASIL-related studies. Collaboration with colorectal surgeons at the hospital was established if simultaneous resection of perianal tissue was needed or if early cancer was suspected.

### 2.2. Data Collection

A semi-structured interview guide was developed by the research team (Appendix A) based on the existing literature, input from healthcare professionals, and frequently asked questions from patients. The interview guide included open questions to allow the patients the opportunity to discuss freely. Interviews were conducted on the hospital premises, although not within the endoscopy unit. The research nurses conducting the interviews were not part of the endoscopy staff but had relevant expertise in the field. Participants were given the option to choose between a digital interview or an in-person interview at the hospital.

A pilot interview was conducted to evaluate the interview guide, which, after discussion in the research team, was included in the study. The interview data were then transcribed verbatim by a professional secretary.

### 2.3. Analysis

The interviews were digitally recorded and saved in an encrypted folder on the hospital server. The transcribed interviews were read several times by the first author PBJ (male doctoral student and nurse endoscopist) to gain clarity regarding the text. The analytical phase was documented in an excel sheet with columns for meaning units, condensed meanings, codes, subcategories and categories.

**Figure 5:**
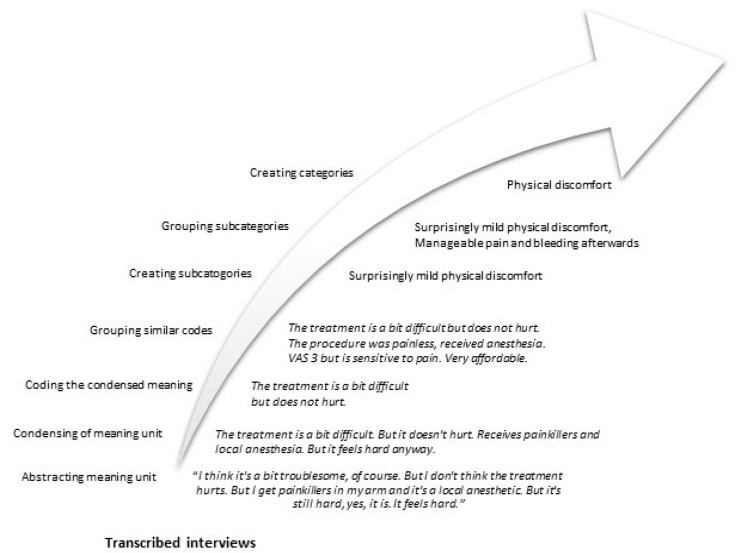
Example of the qualitative content analysis process.

The first author, PBJ, performed the initial abstraction of the text into meaning units and subsequent condensation of the meaning units. These were then compared and discussed with co-authors HD (female RN and PhD) and GD (female RN and PhD), and codes were produced. All codes were printed out on paper and the research team together grouped them into subcategories. All similar subcategories were then collected and documented in tables, which were subsequently posted on a board and re-examined. Similar content was merged to make the data more manageable. Finally, subcategories were grouped into categories (Figure 5). To minimize the risk of important information being lost during translation, subcategories and categories were translated into English after completion of the analysis. Data saturation was assessed through a systematic, iterative, and reflective process, in line with established qualitative research methodologies. After each round of interviews, the research team conducted a thorough review and coding of the transcripts to identify emerging topics and patterns. As the analysis progressed, the appearance of new information diminished. To ensure that saturation had been reached, two additional interviews were conducted to confirm that no new information was emerging. These interviews were independently reviewed by the research team members, and consensus on saturation was reached.

### 2.4. Ethics

Approval for the study was given by the Regional Board of Research Ethics (reference number 2021-00873). All results were anonymized before presentation so that participants cannot be identified.

## 3. Results

A total of 30 invitation letters containing information about the study were distributed to eligible individuals. Of these, 16 requested further details regarding participation. Two explicitly declined to participate, while no response was received from nine individuals. Among those who agreed to participate, two preferred to conduct the interview via a secure digital meeting platform, accessed through an encrypted link. The inclusion and interviews of participants took place from December 2022 to May 2023.

After 14 interviews, data saturation was considered to have been reached. The research group noted that the average interview duration was relatively short. To ensure data saturation, two additional interviews were conducted in September 2024, following the same inclusion criteria as before. These interviews were carried out by the first author (PBJ). No new information emerged from these final interviews, which reinforced the conclusion that data saturation had been achieved.

Nine participants were male, and seven were female. Their mean age was 50.5 years. The interviews lasted for an average of 17 min (range: 10–24 min).

**Figure 6:**
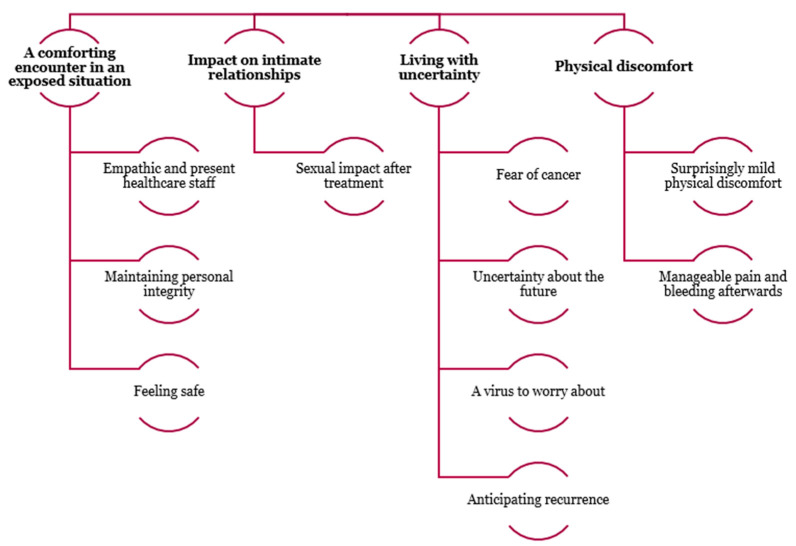
Emerging categories and subcategories from the analysis of semi-structured interviews. The analysis revealed four main categories: 1. a comforting encounter in an exposed situation, with three subcategories; 2. impact on intimate relationships, with one subcategory; 3. living with uncertainty, with four subcategories; 4. physical discomfort, with two subcategories. These categories reflect the participants’ experiences and perceptions, highlighting emotional, relational, existential, and physical dimensions of their situations.

Four categories emerged after the analysis of the semi-structured interviews: a comforting encounter in an exposed situation, with three subcategories; impact on intimate relationships, with one subcategory; living with uncertainty, with four subcategories; and physical discomfort, with two subcategories (Figure 6).

### 3.1. A Comforting Encounter in an Exposed Situation

Living with ASIL, and a possible life-threatening disease, affected the participants in various ways. The participants described a stigma surrounding the disease. The endoscopic treatment was described as uncomfortable and embarrassing, and it was associated with worry about the possible upcoming diagnosis. The participants felt satisfied with their care and that the team had protected their personal integrity. Plans for future controls/treatment were also considered very important.

#### 3.1.1. Empathic and Present Healthcare Staff

Receiving detailed and factual information prior to and during the treatment was important to the participants. Before the examination/treatment, the participants expressed a need for information about ASIL and the treatment procedure. They also appreciated the guidance they received throughout the endoscopic process and treatment. Time given by the team to answering questions and engaging in casual conversations before and after the procedure contributed to a positive experience, with some even describing the situation as enjoyable and humorous.

*I remember being quite nervous at first. It’s some kind of surgery after all. But once I was there, I became very well, the staff were very kind. So, it wasn’t awful at all.*

During the treatment, the participants found the endoscopic team to be calm and focused. They reported that the interaction with the team was important and reassuring, which meant that even if the procedure was experienced as unpleasant and even somewhat painful, it felt acceptable. They were grateful that the endoscopic team was empathetic and that direct action was taken if discomfort occurred during the procedure.

*Well, as I said, it always feels very safe. They explain and I can watch it myself, if I want to, at the same time.*

#### 3.1.2. Maintaining Personal Integrity

The participants felt that protecting their privacy was taken into account, even though they felt exposed and that having their anal canal examined was embarrassing. They understood the necessity of performing the examination and treatment.

*It’s a bit sensitive, being examined like that. But I can honestly say, I anyway felt comfortable in the situation because we had a very good discussion and they were very nice and empathetic.*

Covering paper trousers (with a small opening at the back) helped the participants to feel less exposed; they expressed the importance of privacy, even in front of the endoscopic team.

*I think these trousers help as well. Because I heard about someone who didn’t get them, and then you feel exposed.*

#### 3.1.3. Feeling Safe

The participants experienced a sense of calm and reassurance when the dysplastic lesions were detected and treated. Although worried about recurrence, the participants felt safe because the healthcare system had a follow-up plan and they would be called regularly for check-ups.

*As long as there’s a follow-up, then I can park those thoughts somehow. It’s not something I can control, that’s the way I think.*

Close contact with the endoscopic team and the contact nurse was perceived as important. Participants reported experiencing a sense of security from having the possibility to ask questions about potential complications and future planning.

*And also the nurse I had contact with at the beginning. I still have the piece of paper, I got her number and it felt like oh!*

### 3.2. Impact on Intimate Relationships

Participants experienced stigma surrounding condyloma and ASIL and expressed difficulties talking about it with partners and loved ones. Even though the endoscopic treatment was considered minimally invasive, some participants reported that it had impacted their intimate relationships.

#### Sexual Impact After the Treatment

Some of the participants described how the diagnosis and treatment of anal dysplasia had affected their intimate relationships, especially if pain and a longer period of bleeding occurred after the procedure. In addition, the psychological impact after diagnosis and treatment distanced them from their partner. The perianal region became a more private area, and some avoided exposing this part of their body. Letting someone near that region was perceived as bothersome.

*You don’t want anyone to be near those parts, because it feels uncomfortable. Well, that probably triggers quite a lot of thoughts. Can it be contagious? You know, because it was brought on by warts to begin with. Those thoughts and you really don’t want that. It can definitely be those kinds of thoughts.*

Most participants, however, did not experience any impact on their intimate relationships, even those who had a steady long-term partner. Some even described the discovery and treatment as something good. After treatment, most had waited some time before having penetrative anal intercourse because they wanted to heal. One woman described a feeling of tenderness during vaginal sex. Participants with complications such as pain and bleeding after the procedure waited longer before having sex. However, most participants did not experience sexual problems after the treatment.

*You have to wait X number of weeks for that. Uh I can work that out for myself if you’ve been there and removed it, so I think it should heal, so that hasn’t happened, no.*

### 3.3. Living with Uncertainty

Fear and anxiety were experienced following the diagnosis and treatment of ASIL, with participants describing feelings of uncertainty about the future. They also wondered how long they needed to be worried, whether new ASIL might appear, and how long they would continue being checked and treated. They experienced a sense of insecurity and that life could no longer be taken for granted. Information from the healthcare team made them feel more at ease and the diagnosis less scary. HPV, ASIL relapse, and cancer were all associated with concerns.

#### 3.3.1. Fear of Cancer

Knowledge that anal cell changes may lead to cancer, and that the risk of cancer increases if they are left untreated, was expressed as a concern. ASIL triggered anxiety in participants who had lost a family member or a close friend to cancer. Those with recurrent ASIL and severe dysplasia felt that repeated treatments increased their anxiety. Receiving mail from the hospital also triggered worry.

*It’s the cancer demon. That’s what it is. And it’s quite scary to get anal cancer and colon cancer.*

#### 3.3.2. Uncertainty About the Future

Having ASIL led to participants feeling resigned to the uncertainty. They wondered why they had contracted the disease and when they could relax in the knowledge that ASIL would not return. Participants with relapsing ASIL felt that the treatment did not help. After treatment there was a fear of complications, even if none arose.

*I’m not going to feel safe, because this has come and gone for so long now so I worry, for sure. When can I drop my guard? Is it after one year without symptoms, or after 5 years?*

Participants who received a negative test result found this very difficult and stressful. They experienced the time from the treatment to receiving a test result as long and uncertain. Concern was also created among the participants when the follow-up intervals were increased.

For some, the follow-up could also mean a sense of security, since early lesions could be found in time when they were not yet malignant.

*It’s just magical that there is a method that removes the cell changes before they become malignant, it’s almost too good to be true, I think.*

#### 3.3.3. A Virus to Worry About

Knowledge concerning the relationship between HPV, ASIL, and anal cancer varied among the participants. After being given information, participants described a fear of the virus and the course of the disease. The fact that the virus could create cell changes in the future evoked worry. The participants felt that the virus has a ‘life of its own’ and that it is difficult to get rid of. Questions concerning how long they had had the virus, why they had contracted it, and how long they needed to worry were raised.

*Well, I feel a bit worried about what it is yes, about the virus that it has come back. It has come back every time. Well, the link to cancer can make me worried.*

#### 3.3.4. Anticipating Recurrence

The participants expressed concerns that the dysplasia would return or increase in size between check-ups or treatment sessions. They also expressed a fear that the benign lesions would become malignant. There was a heightened fear of recurrence if the time between controls increased.

*If you think that it keeps coming back, and that it’s good to keep a check on it. I’ve been thinking a bit about it coming back, and that there’s a risk that it could eventually turn into something serious.*

### 3.4. Physical Discomfort

The physical discomfort experienced by the participants occurred both during and after treatment. During the treatment, the main focus was on the perceived pain, while after the treatment, bleeding and pain were the two biggest sources of physical discomfort.

#### 3.4.1. Surprisingly Mild Physical Discomfort

Most participants experienced the treatment as painless, with only some discomfort, and it was described as being easier than expected. They had anticipated that it would be more painful and uncomfortable. Some experienced a minor sting or discomfort when the mucosa was anesthetized, as well as short-term pain if the anesthesia had not fully worked or during treatment in the distal part of the anal canal. Since the endoscopy screen was in front of the participants, they could see if bleeding occurred while anesthetizing the mucosa and during treatment, but this was not perceived as a problem.

*No, but maybe about a 3 [on a numerical rating scale for pain assessment], I think. But then I know that I’m quite sensitive to pain, that I must say, but I found it extremely manageable.*

#### 3.4.2. Manageable Pain and Bleeding Afterwards

The participants described the pain after treatment as short-term, which could be either constant or occurring during defecation. Constant pain was described as a diminishing pain where, in several cases, there was no need for painkillers or anesthetic gel. Some described a more noticeable pain for which anesthetic gel had a satisfactory effect. The pain during and immediately after defecation was described as a minor discomfort/pain. Minor bleeding was common for a few days after treatment; however, if a larger resection had been performed, the postoperative bleeding was sometimes more extensive. Bleeding after treatment was viewed by the participants as something completely normal and not frightening.

*The bleeding is there. The bigger the procedure, the longer the bleeding, but it disappears after a couple of days or a week. So it’s okay.*

## 4. Discussion

In this study, we present novel insights that have been gained from exploring patients’ experiences with a newly introduced endoscopic method for diagnosing and treating ASIL. Although the method itself has been described previously in the literature, it has been briefly outlined in this study for context. ASIL is often recurrent and persons with ASIL are at increased risk of developing invasive cancer. To ensure continued patient adherence to follow-up examinations and treatments, it is essential to understand how patients perceive the endoscopic ASIL treatment.

The participants described the endoscopic treatment as being much easier than expected. Some said the examination was slightly unpleasant, with short-term pain or discomfort experienced when local anaesthetics were injected into the mucosa. They also reported experiencing few complications after the procedure and that the impact on their intimate relationship was low.

However, although the treatment was perceived as gentle and easier than expected, the participants described the condition itself as embarrassing and troublesome. Receiving news about a potentially life-threatening diagnosis created anxiety and a fear of cancer, which led to feelings of uncertainty about the future.

Patients described feeling worried about experiencing pain and discomfort during the endoscopic examination, and the need to have this under control. Literature on the endoscopic treatment of the anal canal is sparse. Pain receptors are activated when the anal canal is examined or treated, which may cause pain and discomfort, and this can affect the overall experience. The endoscopic treatment of the rectum and colon has been described by patients as a somewhat painful and unpleasant procedure [11,14,15,16]. Existing negative attitudes, stories told by friends and acquaintances, social media, and previous experiences are sources of information that may also affect patients’ perceptions of health care [17]. Providing treatment to patients’ most private body areas must be combined with great sensitivity, something that was emphasized by the participants in this study. They appreciated that the endoscopic team treated them with respect and worked in a way that preserved patients’ integrity. Performing an endoscopic examination of the anal canal may be routine for the endoscopic team, a procedure they may perform every day, but for the individual patient it means showing their most private body parts to strangers. For many, this is associated with embarrassment and shame [18,19]. There is also shame and stigma surrounding anal diseases [20]. It is, therefore, of great importance to involve patients and pay attention to how they feel and experience the procedure [21]. The participants in our study expressed the need to be seen and reported that the endoscopic team were responsive to how they experienced the procedure. In Sweden, the majority of endoscopic procedures are performed either without sedation or using conscious sedation techniques. This practice necessitates an endoscopic team proficient in managing stress, anxiety, and stigma. Simulation-based exercises for the entire endoscopy team may provide structured training and guidance for managing real-life clinical scenarios [22,23].

Endoscopy trousers were used to reduce exposure and protect the participants’ integrity. In earlier studies, the use of these has been described as having a positive effect on patients’ experiences of exposure by increasing their integrity [19], and they were greatly appreciated by the participants in our study. Von Wagner et al. described how a patient who undergoes a colonoscopy and receives continuous information regards such informative communication as positive [24]. During endoscopic procedures, patients want conditions such as pain or discomfort to be noticed and remedied by the healthcare staff [25,26].

As well as being given continuous information, the participants also described how a sense of humor from the team helped them through the procedure. Using humor in a natural way has been described as a means of alleviating feelings of embarrassment [24].

Studies have shown that after an endoscopic treatment, patients worry about possible complications, test results, and malignancies [27,28]. Van Heukelom et al. found in their study, comparing treatment of ASIL with imiquimod, topical fluorouracil, or electrocautery, that it was common for patients to experience pain and discomfort, as well as an impact on their sex life, both during the treatment period and up to four weeks after [28]. However, only minor bleeding and pain were reported by the participants in our study, which did not lead to major concern. Some participants described that if the pain was strong, or if a larger resection had been performed that resulted in increased bleeding, their sex life could be affected until the pain and bleeding had stopped. The psychological impact of having anal lesions resulted in some participants not wanting to have sex; however, most described no major impact on their sex life.

The participants reported a psychological impact after endoscopic treatment of ASIL, which they described as “living with uncertainty”. The participants worried about the virus, about recurrence of ASIL, and cancer, which led to feelings of uncertainty about the future.

The participants also described the stigma surrounding ASIL. Questions arose about why the anal cell changes had developed, how long the virus would remain, whether the virus was contagious, and the risk of cancer. Studies have shown that HPV-related lesions affect patients negatively [29]; according to Vriend et al., the negative impact is mostly on an emotional dimension [30]. Mortensen and Larsen described a concern from patients that those around them would perceive them as unclean, careless, or “of easy virtue”—this applied to both men and women. They also described the fear of stigmatization that made the participants not want to inform others about their condition [20]. A fear of rejection due to having a sexually transmitted disease, including HPV-related anal lesions, also existed [31]. The participants in our study described relief from knowing that it was easy to contact the unit and speak to a contact nurse with knowledge about the disease and the post-operative course. Jaensson and colleagues have also confirmed that patient satisfaction increases if nurses are easily accessible [32]. Participants described feelings of joy and security after the treatment and from knowing that there was a plan for follow-up. Previous studies of the experiences of patients treated for anal dysplasia have shown a significant increase in patients’ quality of life after treatment [29,30].

Receiving a potentially life-threatening diagnosis created a fear of cancer, which must be recognized by healthcare professionals. The fear of cancer can be triggered by various factors and may lead to anxiety. Patients want information about how they will be treated and what follow-up may be expected. Waiting for the test result was perceived as troublesome, and the participants wanted to know as soon as possible whether it was cancer or not. Cvejic et al. have described that anal dysplastic test results can cause psychological effects in patients, which can lead to increased cancer anxiety [33]. There is also a fear of social consequences surrounding the disease, as well as the fear of dying. Cancer is seen as something evil, capricious, and an enduring enemy. There are strong links between the fear of cancer and the fear of dying [34,35,36,37]. According to Russo et al., receiving clear communication and information from healthcare professionals helps the patient in dealing with fear and anxiety. In addition, they emphasize that the patient needs to know what the continued care will involve in order to reduce the risk of anal cancer. This strengthens anal health and minimizes the risk of them developing anal cancer; the patient can then experience a sense of control over their health [37].

Although high-resolution anoscopy (HRA) is clinically effective, its wider implementation is limited by access issues, workforce shortages, and inconsistent patient experiences [6]. In a study of 307 MSM, Nowak et al. [38] reported high satisfaction, but also pain and a reluctance to return for a second treatment. Thus, an alternative method to HRA to diagnose and treat anal dysplasia that is better accepted by subjects who have been screened would likely contribute to increased compliance and reduced loss to follow-up.

High-resolution endoscopy of the anal canal offers a promising avenue for expanding the diagnostic and therapeutic capabilities of flexible endoscopy, which is already widely utilized throughout the gastrointestinal tract. Underwater EMR (endoscopic mucosal resection) of the anal canal can be performed by therapeutic endoscopists without the need for longer specialized training. Given the widespread availability of flexible endoscopy equipment and trained personnel in both hospital and outpatient settings, this approach requires minimal additional financial investment.

At our institution, we have conducted a substantial number of endoscopic interventions in patients with ASIL. We are currently undertaking a prospective study aimed at enhancing the endoscopic characterization of dysplastic lesions in the anal canal. This includes detailed assessment of lesion morphology, vascular patterns—specifically capillary architecture and intraepithelial papillary capillary loops (IPCLs)—and lesion localization within the anal canal. The appearance of IPCLs has been previously established as a critical diagnostic marker in esophageal neoplasia.

The strength of the present study lies in its contribution to understanding patient experiences of endoscopic treatment in the early phases of anal dysplasia development, diagnosis, and therapeutic management. This knowledge can be used by healthcare professionals to normalize the treatment and help patients manage the fear and embarrassment associated with the condition. It may also contribute to further development of strategies to help patients cope with the psychological aspects of the treatment and to improve the availability of psychological support when needed. One limitation of this study is the single-center design, with the method currently implemented only at our hospital. As a result, the findings may be influenced by the specific context and practices of our institution. To ensure a balanced representation of both positive and negative experiences, all patients treated from 1 January 2022, onward were invited to participate.

Since the first author of this study worked as one of the endoscopists in the ASIL team, we wanted to avoid feelings of dependence among the study participants. Research nurses were therefore employed to perform the interviews. As the length of the interviews was relatively short, we decided to perform two more interviews to ensure saturation, which were carried out by the first author (PBJ). That the person conducting the endoscopic procedure also interviewed the patients could be seen as a weakness in the study. After analyzing the data, the two interviews confirmed previous content with no new information being added. In the analysis and reporting, the co-authors (who were not based at the endoscopy unit) ensured that objectivity was maintained throughout the process. The COREQ 32-item checklist was used as a guideline to ensure that the study was reported correctly [39].

The study findings underscore the importance of establishing a dedicated care structure for patients diagnosed with anal dysplasia, with particular emphasis on psychosocial support. To ensure more comprehensive care, it is crucial to integrate psychological and psychosocial support services. In a study by Singh and colleagues involving 410 patients diagnosed with STIs, various aspects of psychological health and well-being were examined. The results showed that individuals with genital STIs experienced elevated levels of depression, stress, and a decline in quality of life [40]. A European study also found that STIs have an impact on quality of life, mood, and sexual function. More specifically, symptoms of depression were more common and severe in STIs patients compared to patients with inflammatory chronic diseases involving the anus/rectum, such as ulcerative colitis or other inflammatory bowel diseases (IBD). In addition, sexual functioning was found to be slightly worse in STIs patients than in IBD patients [41]. The emotional and psychological burden of STIs highlights the need for behavioral interventions and supportive care to address stigma, treatment-related concerns, and disruptions in sexual and relational well-being [42]. Nurses play a crucial role in advocating for patients, promoting patient engagement, and contributing to patient safety. Their clinical competence is therefore essential within the endoscopy team to ensure high-quality, safe, and patient-centered care. [43]. This approach facilitates the delivery of individualized counselling tailored to the unique psychosocial challenges faced by patients undergoing treatment for anal dysplasia.

On the basis of our findings, we would like to suggest further studies to explore structured educational interventions to improve patients’ understanding of their condition, treatment options, and coping strategies. We also suggest studies on the provision of psychosocial support, possibly utilizing peer support, which could reduce feelings of isolation and create a sense of community, something that may be particularly helpful among sexual minority populations. This study explored discussions surrounding embarrassment and sexual concerns within a Swedish sociocultural context, which may limit the generalizability of the findings to populations with differing cultural, religious, or sexual norms. Several participants identified themselves as MSM and resided in a metropolitan area where societal attitudes tend to be more accepting of non-normative sexual identities. This urban and socially liberal environment may contribute to participants’ willingness to engage openly in conversations about sensitive topics and may have influenced the participants’ experiences and perceptions. Future research should investigate how these experiences vary across different settings, including rural communities and environments characterized by more conservative cultural or religious values. Additionally, further studies are warranted to explore gender-based differences and to develop tailored interventions aimed at supporting individuals diagnosed with ASIL, particularly in managing anxiety and navigating sociocultural challenges.

## 5. Conclusions

High-resolution flexible endoscopy was described as a gentle and well-tolerated treatment for ASIL, with minimal complications. Given the recurrent nature of ASIL and the likelihood of requiring both multiple follow-up examinations and treatments, a positive patient experience is essential. High-resolution endoscopy enables the extension of the use of flexible endoscopy to the anal canal, including underwater EMR, without endoscopists requiring longer additional training. Given the widespread availability of equipment and expertise, this approach may be cost-effective and readily implementable. When patients perceive the procedure as gentle, their willingness to comply with ongoing care and follow-up is likely to improve, ultimately supporting better clinical outcomes.

However, concerns regarding the virus, recurrence, and cancer were reported. The diagnosis, and the knowledge that ASIL can lead to cancer, evoked anxiety and existential thoughts. Future studies should, therefore, focus on developing support strategies to help patients with ASIL manage their anxiety. This study was conducted within the Swedish healthcare system, where patient autonomy and high trust in medical professionals may shape patient experiences. These contextual factors should be considered when assessing the transferability of findings to other healthcare settings.

## Figures and Tables

**Table 1:** Standardized treatment and follow-up protocol overview.

Lesions Type	Treatment Approach
Isolated ASIL	Initiate primary treatment. If macroscopic radicality is achieved, proceed to surveillance phase.
Multifocal ASIL	Repeated treatments at two-monthly intervals until macroscopic radicality is attained.
**Pathological Outcome**	**Surveillance Interval**
Radical after initial treatment (Isolated) with LSIL	Clinical follow-up at 12 months
Radical after initial treatment (Isolated) with HSIL	Clinical follow-up at 6 months
Radical after repeated treatment (Multifocal lesions)	Clinical follow-up at 6 months
**Subsequent Follow-up Findings**	**Surveillance Interval**
First follow-up: no residual lesion (LSIL)	Schedule control at 12 months
First follow-up: no residual lesion (HSIL)	Schedule control at 6 months
Second follow-up: no residual lesion (LSIL)	Conclude surveillance
Second follow-up: no residual lesion (HSIL)	Schedule control at 12 months

## Data Availability

The data supporting the findings of this study can be obtained from the corresponding authors upon request.

## Appendix A

**Interview guide for a study about the experience of receiving endoscopic treatment for anal cell changes.**

Thank you for agreeing to this interview. Can you tell me how old you are?

1. **Is it correct that you have had cell changes removed endoscopically (i.e., using a flexible instrument that is inserted into the anal canal) here at Ersta hospital?**
2. **Tell me about the time up until the procedure.**
  - What made you contact the healthcare services, for example?
3. **What symptoms did you have before you received the treatment?**
4. **How many treatments have you had?**
5. **What do you know about the treatment that you have received?/What information have you been given about the treatment?**
  - From whom?
  - Found it yourself?
6. **Did you get to choose a treatment option?**
7. **What information were you given about treatment options?**
  - Did you get to choose?
  - How was the decision made?
8. **Do you feel that you were given enough information about the procedure?**
  - If not, what further information would you have wanted?
9. **How did you experience the treatment?**
  - Pain?
  - Bleeding?
  - Privacy?
  - Feeling left out?
  - How were you taken care of?
  - Other problems?
10. **How did you experience the time after the treatment—any problems?**
  - Bleeding? Pain?
  - Difficulty passing stools?
  - Discomfort during sex?
11. **Has anything in relation to the treatment caused you to worry?**
  - Do you want to tell us about your concerns?
  - Fear?
  - If everything has been removed?
  - Cancer?
12. **Has this affected your life?**
  - Your sex life?
  - Your relationship with your partner?
  - Psychological aspects?
  - Anxiety?
  - Worries about the future?
13. **Is there anything that you think we should have discussed but didn’t?**

Thank you very much for your participation.
